# The S-Layer Protein of the Anammox Bacterium *Kuenenia stuttgartiensis* Is Heavily *O*-Glycosylated

**DOI:** 10.3389/fmicb.2016.01721

**Published:** 2016-11-01

**Authors:** Muriel C. F. van Teeseling, Daniel Maresch, Cornelia B. Rath, Rudolf Figl, Friedrich Altmann, Mike S. M. Jetten, Paul Messner, Christina Schäffer, Laura van Niftrik

**Affiliations:** ^1^Department of Microbiology, Institute for Water and Wetland Research, Faculty of Science, Radboud UniversityNijmegen, Netherlands; ^2^Division of Biochemistry, Department of Chemistry, University of Natural Resources and Life SciencesVienna, Austria; ^3^NanoGlycobiology Unit, Department of NanoBiotechnology, University of Natural Resources and Life SciencesVienna, Austria

**Keywords:** anammox bacteria, *Kuenenia stuttgartiensis*, S-layer, glycoprotein, *O-*glycan, methylation

## Abstract

Anaerobic ammonium oxidation (anammox) bacteria are a distinct group of Planctomycetes that are characterized by their unique ability to perform anammox with nitrite to dinitrogen gas in a specialized organelle. The cell of anammox bacteria comprises three membrane-bound compartments and is surrounded by a two-dimensional crystalline S-layer representing the direct interaction zone of anammox bacteria with the environment. Previous results from studies with the model anammox organism *Kuenenia stuttgartiensis* suggested that the protein monomers building the S-layer lattice are glycosylated. In the present study, we focussed on the characterization of the S-layer protein glycosylation in order to increase our knowledge on the cell surface characteristics of anammox bacteria. Mass spectrometry (MS) analysis showed an *O-*glycan attached to 13 sites distributed over the entire 1591-amino acid S-layer protein. This glycan is composed of six monosaccharide residues, of which five are *N*-acetylhexosamine (HexNAc) residues. Four of these HexNAc residues have been identified as GalNAc. The sixth monosaccharide in the glycan is a putative dimethylated deoxyhexose. Two of the HexNAc residues were also found to contain a methyl group, thereby leading to an extensive degree of methylation of the glycan. This study presents the first characterization of a glycoprotein in a planctomycete and shows that the S-layer protein Kustd1514 of *K. stuttgartiensis* is heavily glycosylated with an *O*-linked oligosaccharide which is additionally modified by methylation. S-layer glycosylation clearly contributes to the diversification of the *K. stuttgartiensis* cell surface and can be expected to influence the interaction of the bacterium with other cells or abiotic surfaces.

## Introduction

From early on in the study of Planctomycetes, their cell biology has been a topic that has sparked much interest (Fuerst and Webb, 1991; Fuerst, 1995; Lindsay et al., 1997, 2001). First thought to share a special evolutionary link with eukaryotes (Fuerst, 1995; Devos and Reynaud, 2010; Forterre and Gribaldo, 2010; Fuerst and Sagulenko, 2012), Planctomycetes are now emerging as a special case of Gram-negative bacteria (Speth et al., 2012; Devos, 2014a,b; Jeske et al., 2015; van Teeseling et al., 2015) harboring at least two membrane-enclosed compartments of which the innermost is often characterized by a curved membrane. The width of the periplasmic space in many species varies over the cell volume due to the complex membrane invaginations of the cytoplasmic membrane.

A special case within the Planctomycetes are the anammox bacteria which perform anaerobic oxidation of ammonium with nitrite to dinitrogen gas (Kartal et al., 2011b). Anammox bacteria are present in marine environments, freshwater and soil (Schmid et al., 2007; Dale et al., 2009; Humbert et al., 2010; Zhu et al., 2013; Sonthiphand et al., 2014) where they contribute significantly to the loss of fixed nitrogen. They are also applied in sustainable nitrogen removal systems worldwide (Kartal et al., 2010). Both in nature and in laboratory settings they often occur in clusters in which the cells are attached to other anammox cells and cells of other species (Woebken et al., 2007; Russ et al., 2014). Anammox bacteria can also attach to abiotic surfaces as is exemplified by the common occurrence of biofilms in anammox enrichment bioreactors (Botchkova et al., 2015). Since so far anammox bacteria cannot be grown in pure culture, all anammox species officially have a “*Candidatus*” status. The anammox cell plan comprises a third membrane-enclosed compartment called anammoxosome (Lindsay et al., 2001). This prokaryotic organelle is the location of the anammox reaction that supports the energy for growth of these bacteria (Neumann et al., 2014). In contrast to other planctomycetes, it is the membrane of the anammoxosome instead of the cytoplasmic membrane that is curved. In this case the membrane curvature is hypothesized to provide an enlarged surface area for the many membrane-associated proteins involved in the anammox reaction (van Niftrik et al., 2004; van Teeseling et al., 2013).

The cell envelope of Planctomycetes has been an object of many studies. Early studies (in non-anammox Planctomycetes) reported the almost ubiquitous macromolecule peptidoglycan to be absent and proteinaceous cell walls to be present instead (König et al., 1984; Liesack et al., 1986; Cayrou et al., 2010). Recently, it was discovered that both non-anammox (Jeske et al., 2015) and anammox (van Teeseling et al., 2015) Planctomycetes do have a peptidoglycan cell wall. In addition, the model species “*Candidatus* Kuenenia stuttgartiensis” (referred to as *K. stuttgartiensis* throughout the manuscript) was found to have a proteinaceous two-dimensional crystalline surface (S-) layer (van Teeseling et al., 2014). S-layers have been found in almost all phylogenetic branches of Gram-positive and Gram-negative Bacteria as well as in Archaea (Fagan and Fairweather, 2014), but this was the first report in a cultured planctomycete. It is generally assumed that S-layers provide a selection advantage to the cells, by one or several of the reported functions: osmoprotection (Engelhardt, 2007b), maintenance of the cell shape and integrity (Engelhardt, 2007a; Klingl et al., 2011), protection against predation (Tarao et al., 2009; Chanyi et al., 2013) and attachment of exoenzymes (Egelseer et al., 1995). S-layers are formed by the intrinsic self-assembly capability of the constituting S-layer (glyco)protein monomers. Covalently linked glycans are often found in S-layers and these glycans are highly diverse in terms of composition and structure (Messner et al., 2013). The *K. stuttgartiensis* S-layer is composed of many copies of the modified protein Kustd1514 that has an apparent molecular mass of 250 kDa and also occurs in unmodified form (160 kDa), albeit to a lesser extent.

Since the S-layer is the outermost cell envelope layer and therefore, the structure that is in contact with the outside environment it is expected to have an important function for the cells. This hypothesis is strengthened by the observation that *K. stuttgartiensis* cells have not lost their S-layer during prolonged culturing under laboratory conditions – even though this is a frequent observation for other S-layer bearing bacteria (Sleytr and Messner, 1983). Because of the lack of a genetic system in anammox bacteria, it was not possible to test the importance of the S-layer via a knock-out mutant. In this study, we set out to characterize this S-layer in more detail, focusing on the modification of the S-layer protein. Previously, the detection of the 160-kDa S-layer protein with the carbohydrate-specific periodic acid Schiff (PAS), stain migrating at an apparent molecular weight of ∼250 kDa, indicated this protein to be glycosylated (van Teeseling et al., 2014). In this study, we performed a detailed mass spectrometry (MS) analysis to find out how the glycans are attached to the S-layer protein, which sugars constitute the glycan and if and how these sugars are further modified.

## Materials and Methods

### *K. stuttgartiensis* Enrichment Culture

Free-living planktonic *K. stuttgartiensis* cells were grown in an enrichment culture (∼95% *K. stuttgartiensis*) in a membrane bioreactor as described previously (Kartal et al., 2011a).

### Preparation of *K. stuttgartiensis* S-Layer Glycoproteins

The S-layer was enriched from *K. stuttgartiensis* cells concentrated in their original growth medium (van de Graaf et al., 1995) which were frozen at -20°C. Thawed cells were resuspended in 20 mM potassium phosphate buffer pH 7 with 750 mM 6-amino caproic acid, and broken in a French Press (three passages at 138 MPa). The membrane fraction, which contains the S-layer as one of the most abundant proteins, was collected after ultracentrifugation (184000 *g*, 60 min). This enriched S-layer protein sample was washed three times in the above-mentioned buffer and stored until further use.

### PNGaseF Treatment of S-Layer Protein

To release putative *N*-glycans, S-layers, enriched as described before (van Teeseling et al., 2014), were incubated with peptide-*N*-glycosidase F (PNGaseF; ∼0,25 units of PNGaseF per μg of enriched S-layer protein) at 37°C for 10 h in 20 mM potassium phosphate buffer pH 7. As a negative control, enriched S-layer protein was incubated without PNGaseF. After incubation, the samples were analyzed via SDS-PAGE on 8% slab gels using Laemmli running buffer (Laemmli, 1970) followed by staining with Coomassie Brilliant Blue G250.

### MS/MS Analysis of S-Layer Glycopeptides

MS/MS was performed on tryptically digested S-layer glycoprotein bands excised from an SDS-PAGE gel to which enriched S-layers (described above) were applied following the protocol as described before (Kolarich et al., 2012). In brief, bands were destained, S-alkylated and digested with sequencing grade trypsin (Promega, Vienna, Austria). The peptide mixture was analyzed using a Dionex Ultimate 3000 system directly linked to a QTOF instrument (maXis 4G, Bruker) equipped with the standard ESI source in the positive ion, data-dependent mode. MS-scans were recorded (range: 150–2200 m/z, spectra rate: 1 Hz) and the six highest peaks were selected for fragmentation. Instrument calibration was performed using an ESI calibration mixture (Agilent). For separation of the peptides, a Thermo BioBasic C18 column (5 μm particle size, 150 mm × 0.36 mm) was used. A gradient from 97% solvent A (65 mM ammonium formate) and 3% solvent B (100% acetonitrile) to 32% solvent B in 45 min was applied at a flow rate of 6 μl/min. Analysis data was converted to XML files and evaluated against the target sequence using X! Tandem^1^ with the following settings: reversed sequences no; check parent ions for charges 1, 2, and 3 yes; models found with peptide log e lower -1 and proteins log e lower -1; residue modifications; oxidation on M, W and deamidation of N, Q; isotope error was considered; fragment type was set to monoisotopic; refinement was used with standard parameters; fragment mass error of 0.1 Da and ± 7 ppm parent mass error; fragment types b and y ions; maximum parent ion charge of 4; missed cleavage sites allowed was set to 2; semi-cleavage yes. The MS^2^ trace was manually screened for ions of tryptic peptides derived by complete loss of sugars from the parent glycopeptide.

### RP-HPLC Detection of Monosaccharide Components of the S-Layer Glycan

To determine the monosaccharide composition of the glycan attached to the S-layer glycoprotein, S-layer glycoprotein bands were excised from SDS-PAGE gels, destained and incubated with pepsin (Sigma–Aldrich, P6887) in 5% formic acid. Extracted material was dried, resuspended in 4 M trifluoroacetic acid and hydrolyzed at 100°C for 4 h. The resulting monosaccharides were labeled with 2-aminobenzoic acid (Anumula, 1994) and analyzed by RP-HPLC using a volatile buffer system (Windwarder et al., 2016).

## Results

### PNGaseF Treatment of *K. stuttgartiensis* S-Layer Glycoprotein

**FIGURE 1:**
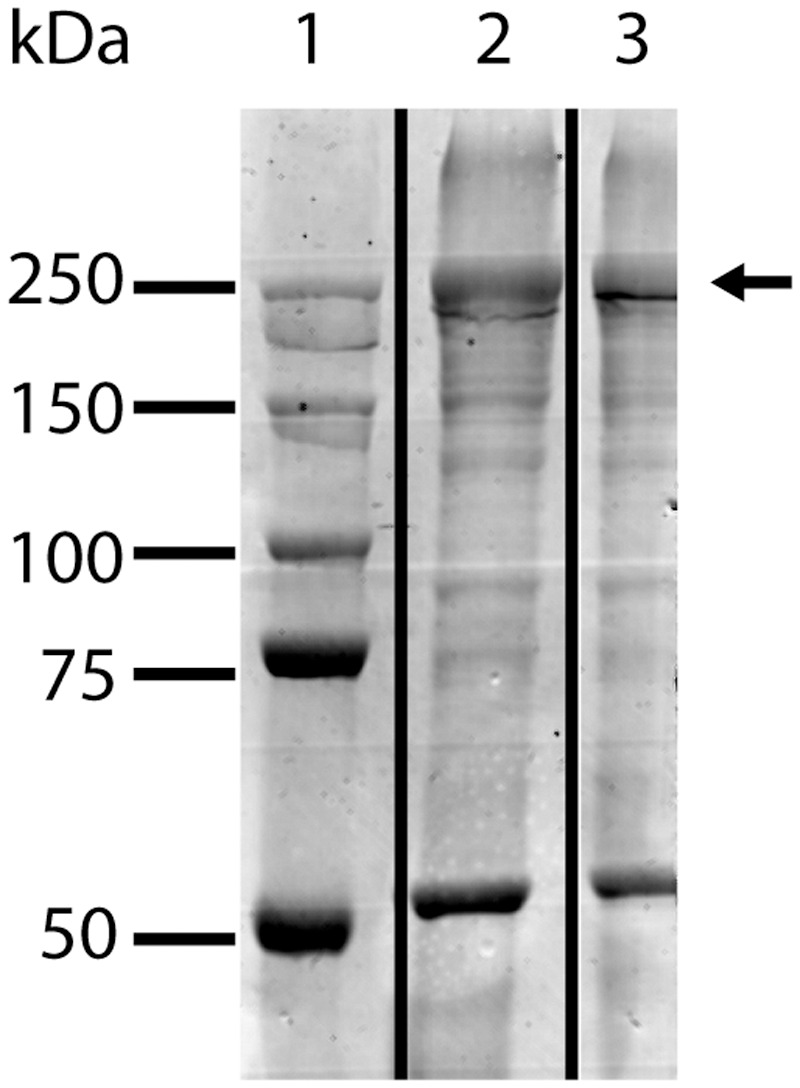
**SDS-PAGE analysis of *K. stuttgartiensis* S-layer glycoprotein enrichment incubated for 10 h at 37°C with (lane 2) and without (lane 3) PNGaseF.** PNGAseF treatment did not alter the migration behavior of the S-layer glycoprotein indicating that no *N*-linked glycans were cleaved off the S-layer glycoprotein (indicated with the arrow). Marker with molecular mass standards in lane 1.

Previous results suggested that the S-layer protein Kustd1514 is a glycoprotein, since the carbohydrate-specific PAS stain colored the Kustd1514 band at approximately 250 kDa strongly (van Teeseling et al., 2014). To verify these results and to test if the S-layer glycans were linked to the protein via an *N*-glycosidic bond, the enriched S-layer glycoprotein was treated with PNGaseF. This enzyme specifically releases *N*-linked glycans from the protein by cleaving the amide linkage between asparagine and oligosaccharides with a chitobiose core. SDS-PAGE demonstrated that incubation with PNGaseF did not cleave the glycoprotein, since the pattern on an SDS gel was identical to that of the untreated sample (**Figure 1**). This result indicated that, provided the S-layer protein is indeed a glycoprotein, the glycan is most probably *O*-glycosidically linked to the protein.

### MS/MS Analysis of Tryptic (glyco)peptides from the S-Layer Glycoprotein Kustd1514

**FIGURE 2:**
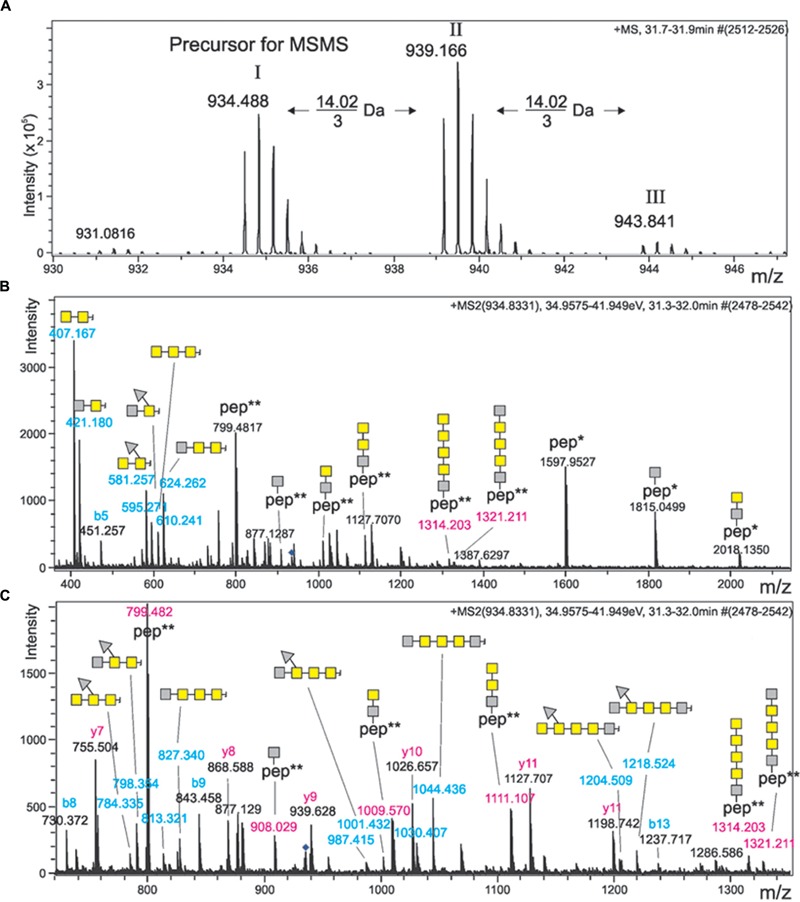
**Mass spectrometric analysis of the *K. stuttgartiensis* glycopeptide prepared in this study. (A)** Shows the sum spectrum of the triply charged ion of glycopeptide LGTQATSALPLIALTK, which comes in three versions spaced by 14.02/3 Da. **(B,C)** Depict the same MS/MS spectrum of compound I with different *x*-axis. Note that the precursor mass selection window was chosen large enough to co-select peak II. Mass values printed in blue depict B-ions of the non-reducing end of the glycan. Glycopeptide masses (Y-ions) are written in red, whereas the peptide-only fragment masses are given in black. Yellow squares denote *N*-acetylgalactosamine residues, while gray squares stand for a methylated HexNAc. Gray triangles represent di-methylated deoxyhexose. ^∗^ and ^∗∗^ indicate the charge state of peptide ions.

**FIGURE 3:**
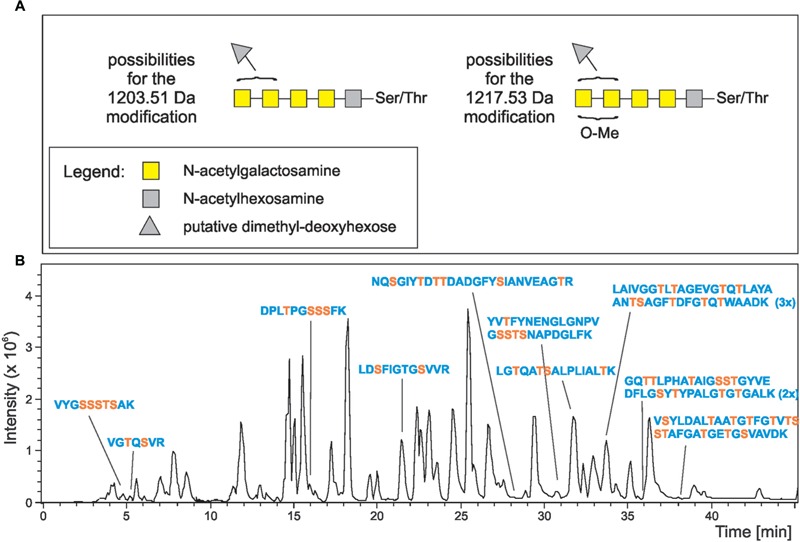
**Structure model of the *K. stuttgartiensis* S-layer glycoprotein *O*-glycan and its occurrence. (A)** Summarizes our current view of the *O*-glycan on the Kustd1514 S-layer glycoprotein. As the exact location of the 174-Da unit and the methyl group are not known, several isomeric structures are possible. The brackets indicate the possible locations of the methyl group (*O*-Me) and the 174-Da component (gray triangle). Again, yellow squares denote *N*-acetylgalactosamine residues, while gray squares stand for a methylated HexNAc. **(B)** Shows the base peak ion chromatogram of the tryptic digest of *K. stuttgartiensis* S-layer glycoprotein. The SDS-PAGE band of the protein Kustd1514 was S-alkylated, digested with trypsin and subjected to reversed-phase HPLC with mass spectrometric detection. Peaks of peptides found to carry one or more of the *O*-glycan units found on LGTQATSALPLIALTK are designated. Serine and threonine residues are depicted in orange.

The ∼250-kDa band that was shown before to represent modified Kustd1514 (van Teeseling et al., 2014) was excised from an SDS-gel, digested with trypsin and subjected to mass spectrometric analysis. ESI-MS/MS spectra of mammalian *N-* and *O-*glycoproteins are often characterized by the occurrence of a peak for the peptide ion plus one HexNAc residue (“Y_1_ ion”) (Irungu et al., 2008). Likewise, oxonium ions of HexNAc and oligomers are usually found. However, these strategies were not so suitable for this sample because no prior knowledge was present on the glycan composition. Indeed these strategies did not identify signals belonging to glycopeptides. However, a signal was noticed where a double peak with a 14-Da mass difference was seen. This could indicate *O*-methylation of a glycan. The base peak of the fragment spectrum had the mass of the doubly charged peptide LGTQATSALPLIALTK and in fact the spectrum contained a series of y and b fragments confirming the identity of this peptide (**Figure 2**). Notably, this Kustd1514 peptide does not contain any Asn residue and hence the modification can be designated as an *O-*glycan, confirming the PNGaseF results. The glycan found on this peptide gave rise to a mass increment of 1203.507 Da, which could not be readily explained in terms of plain sugar units. However, the fragment at m/z 908.029 was followed by two more fragments spaced by m/z 101.54 (=203.07/2) as is typical of HexNAc residues. The gap to the naked peptide mass at m/z 799.48 amounted to a mass indicative of an *O-*methylated HexNAc. The picture was completed by ions from the non-reducing end of the sugar moiety (B-series) starting with m/z = 407.17, which comprises two HexNAc units. This B_2_ ion is then elongated either by m/z 203.07 (=HexNAc) or by a residue with m/z 174.09, which could be a di-methylated deoxyhexose or –even more exceptional- a trimethylated pentose that sits on the terminal or penultimate HexNAc of the non-reducing end. The parent ion of this glycopeptide was accompanied by a peak that was larger by m/z 4.67 (=14.02/3 Da), which indicates an additional methyl group. The MS/MS spectrum, which because of the selected window size comprises fragments of both these precursors, indicated that this additional methyl group is attached to the terminal HexNAc disaccharide. Together with the monosaccharide analysis (described below), which identified the HexNAc component as a GalNAc, the model shown in **Figure 3** was devised. A survey of the elution region of this *O-*glycopeptide indicated it as being the dominant, if not only, glycoform of Kustd1514.

### RP-HPLC Detection of Monosaccharide Components of S-Layer Glycoprotein

Since there is no mass difference between sugar epimers, MS cannot distinguish between different HexNAc or dHex sugars. Therefore, we analyzed the glycopeptides used for MS analysis as 2-aminobenzoic acid derivatives with a volatile buffer system for RP-HPLC that allowed verifying the nature of peaks by MS (Windwarder et al., 2016). Three major peaks were observed. The largest peak was identified as glucose, which most likely was as contamination introduced with the pepsin. Another peak with the mass of a HexNH_2_ exactly eluted like GalNH_2_. The third peak did not co-elute with any of the available standards and had the mass of a mono-methylated HexNAc. No peak with the mass accounting for the 174 Da component in the MS/MS spectra could be found in the time window considered.

### MS/MS Analysis Showed Glycosylation Sites at the S-Layer Protein

**FIGURE 4:**
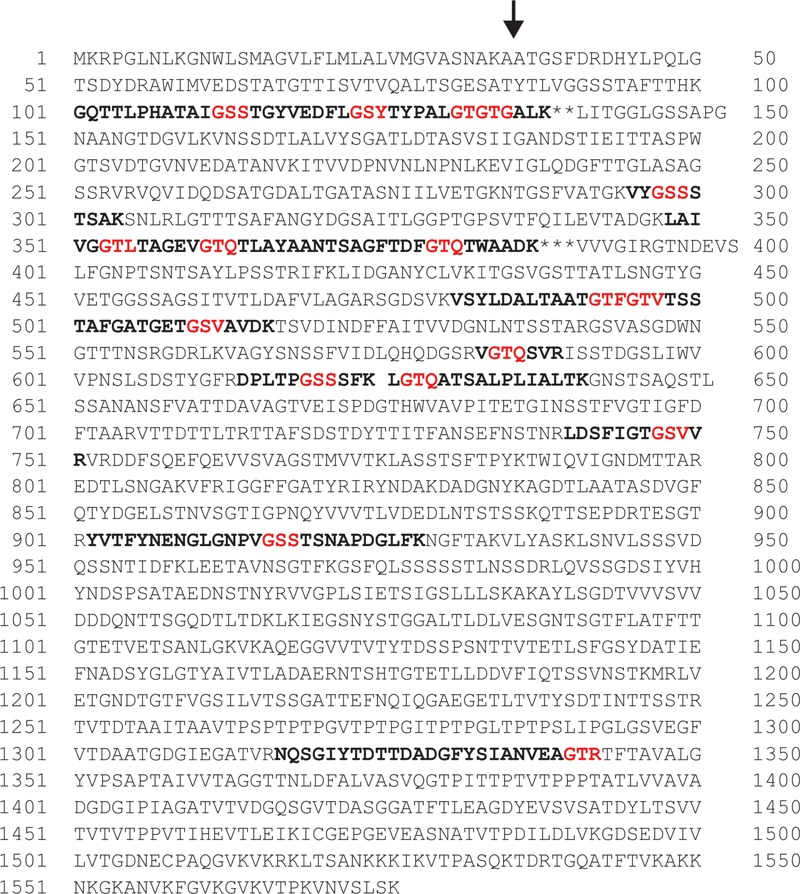
**Amino acid sequence of the S-layer glycoprotein Kustd1514 with glycopeptides detected via MS in bold and putative glycosylation recognition sites in these peptides in red.** The peptide followed by ^∗∗^ was found with two glycans attached, the peptide followed by ^∗∗∗^ was carrying three glycans. The putative amino acid cleavage site of the signal peptidase is indicated with an arrow. The blank space in line 601 is inserted to indicate that these two glycopeptides on both side of the blank space have both been identified.

Further inspection of the MS/MS data revealed a total of ten peptides carrying the described *O-*glycan (**Figure 3**). One of these peptides carried two copies of the glycan, while another peptide was found to contain three copies of the glycan. Thus, a total of 13 different sites at the S-layer protein was identified that carry this *O-*glycan. The majority of the *O-*glycans was attached to the N-terminal half of the 1576-amino acid protein and only two glycans were found to be attached to the C-terminal half (**Figure 4**).

## Discussion

The cell envelope has been a crucial structure in interpreting the controversial planctomycetal cell plan. Only recently peptidoglycan was revealed in several planctomycetes (Jeske et al., 2015; van Teeseling et al., 2015) and a proteinaceous S-layer was described in the anammox bacterium *K. stuttgartiensis* (van Teeseling et al., 2014). As at the time of the discovery of the S-layer, the peptidoglycan layer in these bacteria had not yet been found, it was hypothesized that the S-layer provided integrity and strength to the anammox cells (van Teeseling et al., 2014). Now it is known that the S-layer is not the only cell wall component and even though it cannot be excluded that the S-layer is still necessary in providing additional strength and integrity as an exoskeleton (as suggested by Engelhardt, 2007a), it is also well possible that the S-layer has a different function for *K. stuttgartiensis*. By describing an *O-*glycan that is present in the cell envelope of the anammox bacterium *K. stuttgartiensis*, the present study adds to a more in-depth knowledge of planctomycetal cell envelopes and shows that glycoproteins can make up an important part of these structures.

The *O*-glycan described in this study was found attached to the *K. stuttgartiensis* S-layer protein Kustd1514 at thirteen different sites on ten different peptides. With the methods used it was not possible to identify at which amino acids within these peptides the glycans were attached. Knowing that the glycan is an *O*-glycan, we searched for serine and threonine residues in these peptide sequences, since the glycans are expected to be bound to these residues. The identified peptides all harbor a serine and all but one contain threonine residues. Therefore, we can conclude that the glycan at least in multiple of the cases attaches to the serine residue. However, not all glycans can bind to a serine, since the peptide that carries the triple glycosylation has only one serine and therefore two of the three glycans can be assumed to be bound to a threonine. It seems most likely that the glycan is always attached to the protein via an *O-*linkage, even though some asparagine residues were observed in the glycopeptides. This also fits with previous findings of bacterial S-layer proteins that are exclusively glycosylated via *O-*linkages, even though some other glycoproteins in Bacteria and S-layer glycoproteins in Archaea can show *N-*glycosylation (Ristl et al., 2011; Jarrell et al., 2014).

Glycosylation sites are often characterized by a specific sequence that is recognized by the enzyme that couples the glycan to the protein, either as an entire glycan coupled via an *O-*oligosaccharyltransferase (*O-*OTase) or monosaccharide by monosaccharide in an *O-*OTase independent pathway (Iwashkiw et al., 2013). Since the glycopeptides that were identified with MS analysis were rather long and only one glycoprotein was analyzed it is difficult to point to a recognition sequence for *O-*glycosylation in *K. stuttgartiensis*. However, analysis of the glycopeptides showed seventeen potential glycosylation sites where the serine or threonine is preceded by a glycine (**Figure 4**). Since in each glycopeptide the sequence GT/SX (in which X is variable) is present at least once (and at least twice in the peptide carrying two glycans and at least three times in the peptide carrying three glycans), it seems probable that GT/SX is the recognition sequence. Since this sequence is also present on multiple sites in the S-layer protein outside of the identified glycopeptides, additional clues are probably present that steer the glycosylation toward the sequences inside the glycopeptides. In some bacteria, the same (*O*-)glycan can be coupled to several (often cell envelope-associated or excreted) proteins (Nothaft and Szymanski, 2010; Posch et al., 2011; Iwashkiw et al., 2013) and it remains to be investigated if the described glycan is also present at other proteins of *K. stuttgartiensis*.

Since almost no unglycosylated variants of the glycopeptides were found, it seems that most of the thirteen identified glycosylation sites are occupied by glycans in one and the same mature glycoprotein. Therefore, it can be concluded that the amount of glycosylation is rather exceptional for Bacteria. Another indication for a high degree of glycosylation of the S-layer protein is the large difference between the apparent molecular mass of the glycosylated (250 kDa) and native protein (160 kDa). The nominal mass of all glycans described in this study (∼1.2 kDa in case of full glycosylation of the thirteen identified sites) cannot explain this difference. However, glycosylation often leads to pronounced, unexpected shifts in apparent molecular mass (Magnelli et al., 2011), as it affects the binding of SDS. Furthermore, *O*-glycosylation sites tend to be located in more rigid regions of a protein, which leads to inappropriate calibration with globular proteins (Léonard et al., 2005, 2010). In Bacteria, the number of glycosylation sites per protein is often below 10 (Iwashkiw et al., 2013) and most of the known S-layer proteins have been described to have two to four glycosylation sites per S-layer protein (Ristl et al., 2011). In archaeal S-layers, a higher amount of glycosylation sites has been described. In the case of the *N-*glycan of the archaeon *Sulfolobus acidocaldarius* S-layer protein, an exceptionally high number of 45 potential glycosylation sites has been described, of which nine have been experimentally verified to carry a glycan (Peyfoon et al., 2010).

The *O-*glycan described in this study consists of six monosaccharides and is shorter than most bacterial S-layer glycans, which can contain up to 150 monosaccharides (Messner et al., 2008). Almost all *O-*glycans coupled to bacterial S-layers contain a repeating unit that is linked to the protein via a core oligosaccharide (Messner et al., 2008; Ristl et al., 2011; Fagan and Fairweather, 2014), with the so far only exception provided by the *O-*linked oligosaccharide decorating the S-layer proteins of *Tannerella forsythia* (Posch et al., 2011). The *K. stuttgartiensis* S-layer *O-*glycan has no repeating units and, thus, shares its S-layer glycan building plan with *T. forsythia*. The presence of methyl groups in the *K. stuttgartiensis* S-layer is in itself not exceptional, since methylation has been described before for S-layer glycans in both Bacteria (Schäffer et al., 1999, 2002; Messner et al., 2008; Posch et al., 2011) and Archaea (Paul and Wieland, 1987; Kärcher et al., 1993; Magidovich et al., 2010), as well as in Eukaryotes (Staudacher, 2012). With up to five methyl groups in six monosaccharides, the degree of methylation of the *K. stuttgartiensis* S-layer glycan is however, higher than has been described for bacterial S-layer glycans. In most other bacterial S-layer glycans methylation has thus far only been described for the terminal sugar residue at the non-reducing end (Messner et al., 2008). In the glycan described here at least one of the methyl groups is present at another site, being the reducing-end sugar residue (**Figure 3**).

The specific identity of the methylated HexNAc could not be clarified via monosaccharide analysis since no methylated HexNAc standards were included in the analysis. Since, however, one of the two methylated HexNac residues was found in an unmethylated state as well (**Figures 2B,C**) and no other unmethylated HexNAcs were found other than GalNAc, it seems most probable that this HexNAc is a GalNAc which is methylated in some cases. Also the 174-Da component could not be identified with the RP-HPLC approach, since no peak was observed that could stem from this component. Maybe the peak stemming from this component eluted outside of the observed region or the amount was too low to be detected. At this point it remains to be elucidated if the 174-Da component is indeed a dimethylated deoxyhexose, a trimethylated pentose or even something else. Dimethylated deoxyhexose, however has been found in multiple bacteria before (Takaichi et al., 2001; Schorey and Sweet, 2008).

An intriguing question concerning the glycosylation of Kustd1514 is which effect on, and which function for, the cells these glycans have. Such an extensive glycosylation is expected to have an impact on the physicochemical properties of the S-layer, and thereby the interface of the cell that is seen by the extracellular environment. Indeed, glycosylation was shown to increase the hydration of the S-layer protein in the bacterium *Geobacillus stearothermophilus* (Schuster and Sleytr, 2015). Possibly the glycosylation of Kustd1514 protects the protein from protease degradation, since this was shown in the case of *N-*glycosylation of S-layer proteins in Archaea (Yurist-Doutsch et al., 2008, 2010; Kaminski et al., 2010) and several bacterial non-S-layer *O-*glycans (Herrmann et al., 1996). Another described role of protein glycosylation in Bacteria is attachment, for instance to eukaryotic cells (Swanson and Kuo, 1994; Lindenthal and Elsinghorst, 1999) and cellulose (Miron and Forsberg, 1999). Since attachment to other cells and abiotic surfaces is a common characteristic of anammox bacteria it could well be that the glycosylation of the S-layer plays a role in attachment. Since in anammox bacteria no genetic system is present and attempts to grow anammox cells in pure culture have not succeeded up to now, it will be very difficult to test the role of the S-layer glycans.

In summary, this study describes an *O-*glycan that is linked to the S-layer protein of the anammox bacterium *K. stuttgartiensis*. The *O-*glycan is composed of six monosaccharide residues, and built up of five HexNAc residues – of which four have been confirmed to be GalNAc. One of these HexNAcs is always methylated and a second one is methylated in some cases. In addition a putative dimethylated deoxyhexose completes the glycan. Compared to most other structurally elucidated bacterial S-layer glycans, this glycan is shorter, shows an extensive degree of methylation and is linked to many different sites of the protein. This study deepens the understanding of the cell envelope of the anammox Planctomycetes and provides the first description of a planctomycetal glycoprotein.

## Author Contributions

All listed authors have made substantial, direct and intellectual contribution to the work, and approved it for publication.

## Conflict of Interest Statement

The authors declare that the research was conducted in the absence of any commercial or financial relationships that could be construed as a potential conflict of interest.

## Footnotes

1 www.thegpm.org/tandem/
